# Differences in age at diagnosis of breast cancer largely attributable to different age structures

**DOI:** 10.1093/oncolo/oyag292

**Published:** 2026-07-31

**Authors:** Talía Malagón

**Affiliations:** Department of Oncology, McGill University, Montréal, QC H4A 0B3, Canada; St Mary’s Research Centre, CIUSSS de l‘Ouest-de-l’Île-de-Montréal, Montréal, QC H3T 0A2, Canada

I read with great interest the article by Wilkinson et al., “Breast cancer incidence and mortality, by age, stage, and molecular subtypes, by race/ethnicity in Canada”.^1^ This article addresses a dearth of data stratified by race and ethnicity in Canada, and its reporting of age-specific incidence and mortality rates by race and ethnicity is a very important contribution to the literature. This data shows that incidence rates substantially vary by race and ethnicity in Canada and can help inform research into the etiology and prevention of breast cancer.

However, I find the analysis of age at breast cancer diagnosis presented in their Figure 1 to be problematic, especially as this is the basis on which the authors provide their main conclusion that the peak age at breast cancer diagnosis is younger in non-White racial and ethnic groups compared with White women. It is very clear that the age at breast cancer diagnosis is strongly influenced by the different age distributions of each racial and ethnic group. The older age at diagnosis of White women is largely due to the fact that White women in Canada are substantially older than women from other racial and ethnic groups, as shown in Table 1 of Wilinson et al. While this is mentioned by the authors as a potential explanation, I am disappointed that they did not attempt to adjust for this difference in age structure in any way. This means that the comparison of ages at diagnosis between groups is not a fair comparison and does not inform us very much about the underlying risk of breast cancer for women from different groups at the same age. This is equivalent to comparing crude incidence rates between populations with different age structures, and the reason why incidence rates are age-standardized for comparisons of different populations. Differences in age structure are not modifiable and are generally not inherently interesting as a target for cancer control.

**Figure 1.**
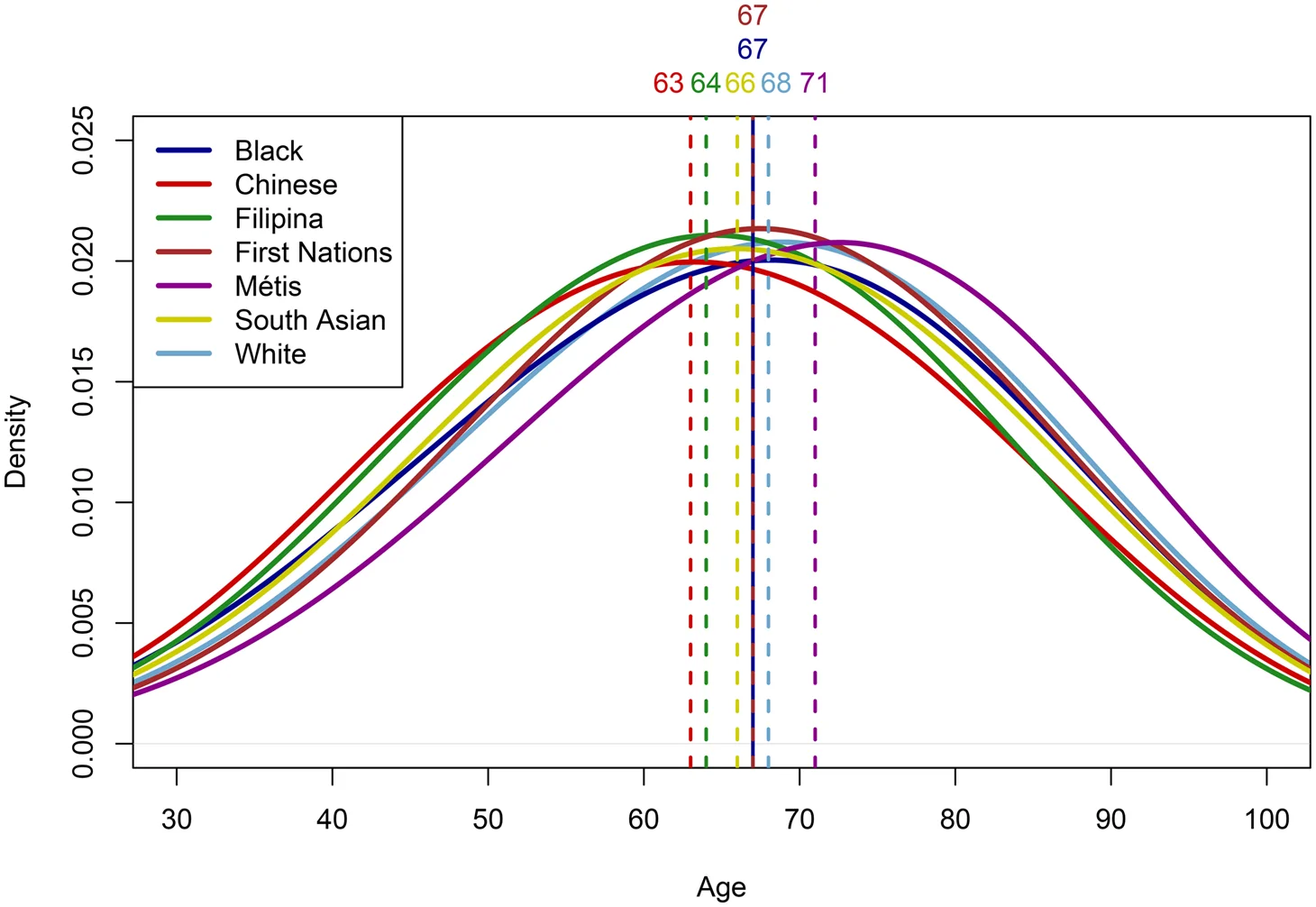
Distributions of age at breast cancer diagnosis, by race/ethnicity, age-standardized to 2021-2023 female Canadian life tables. The incidence of breast cancer at each age was calculated by multiplying age-specific incidence rates of each racial and ethnic group with the number of life years lived at each age in the life table. Due to lack of data for ages <30 years, it is assumed all racial and ethnic groups experience the same incidence rate as women <30 years reported for all of Canada. Results are smoothed using kernel density smoothing. Vertical dashed lines indicate the age-standardized median expected age at diagnosis for each group.

It is fairly straightforward to control for differences in age structure when looking at age at diagnosis by standardizing the cumulative risk to a standard cohort.^2^ Using the age-specific incidence rates reported by Wilkinson et al. in their Table 2, I have age-standardized the cumulative lifetime risk of breast cancer in each racial and ethnic group using person-years from the 2021-2023 Canadian life tables,^3^ and plotted the density of diagnoses by age (Figure 1). Figure 1 can be interpreted as the distribution of breast cancer diagnoses by age in a hypothetical cohort assuming each racial and ethnic group has equal life expectancy or, alternatively, as breast cancer cases by age assuming all groups have the same age structure. It is evident that adjusting for age structure accounts for a large part of the differences in age at diagnosis present in the original crude analysis, and the expected age-standardized median age at diagnosis of breast cancer in the CanCHEC cohorts by the end of their lifetimes would generally center in the mid-sixties for most racial and ethnic groups, with the exception of Métis women, whose age-standardized median age at diagnosis is expected to be older at 71.

Because most people only read the abstract, unfortunately the main takeaway that most readers will have from this paper will be that non-White women are diagnosed with breast cancer at younger ages, when a more nuanced interpretation is warranted. Namely, that while there are differences in risk of breast cancer by age by race and ethnicity, differences in age at diagnosis today largely reflect differences in the age structure of different racial and ethnic groups in Canada.

## References

[1] Wilkinson AN, Ng C, Ellison LF, Seely JM. Breast cancer incidence and mortality, by age, stage and molecular subtypes, by race/ethnicity in Canada. Oncologist. 2025;30:oyae283. 10.1093/oncolo/oyae283 39487041 PMC12395137

[2] Sasieni PD, Adams J. Standardized lifetime risk. Am J Epidemiol. 1999;149:869-875. 10.1093/oxfordjournals.aje.a009903 10221324

[3] Statistics Canada. Life Tables, Canada, Provinces and Territories 1980/1982 to 2021/2023 (three-year estimates), and 1980 to 2023 (single-year estimates)Life tables, Canada, provinces and territories, catalogue no. 84-537-X. 2024. Accessed October 07, 2025. https://www150.statcan.gc.ca/n1/pub/84-537-x/84-537-x2024001-eng.htm

